# Longitudinal Course of Gait Kinematics and Kinetics After Guided Growth for Idiopathic Genu Valgum: Three-Dimensional Gait Analysis to Skeletal Maturity

**DOI:** 10.3390/jcm15207898

**Published:** 2026-10-13

**Authors:** Philipp Scheider, Ghanem El-Isa, Sebastian Farr, Andreas Kranzl

**Affiliations:** 1 Department of Pediatric Orthopaedics and Foot and Ankle Surgery, Orthopaedic Hospital Speising, 1130 Vienna, Austria; ghanem.el_isa@hotmail.com (G.E.-I.); sebastian.farr@oss.at (S.F.); 2 Medical Campus, Medical University of Vienna, 1090 Vienna, Austria; 3 Laboratory for Gait and Movement Analysis, Orthopaedic Hospital Speising, 1130 Vienna, Austria

**Keywords:** pediatric orthopedics, three-dimensional gait analysis, gait biomechanics, knee kinematics, knee kinetics, transverse-plane gait, longitudinal follow-up

## Abstract

**Background/Objectives:** Guided growth changes frontal-plane gait mechanics in idiopathic genu valgum, but the durability of these adaptations through skeletal maturity remains uncertain. This study examined their long-term course. **Methods:** Seventeen participants (31 treated limbs) underwent three-dimensional gait analysis before plate insertion (t0), shortly before plate removal (t1), and after skeletal maturity (t2). Primary outcomes were the mean frontal knee angle during single support and the early- and late-stance peaks and stance mean of the internal frontal-plane knee moment. Longitudinal changes were assessed using linear mixed-effects models accounting for participant and limb dependence. Exploratory comparisons used laboratory reference data from 11 healthy participants. **Results:** Mean follow-up after plate removal was 59.66 ± 12.13 months. The mean frontal knee angle changed from −5.34° ± 2.61° at t0 to −0.08° ± 2.66° at t1 and −0.61° ± 2.27° at t2. The model-estimated change from t1 to t2 was −0.53° (95% confidence interval, −1.50° to 0.44°; Bonferroni-adjusted *p* = 0.798). All three knee-moment measures increased from t0 to t1, with no significant further changes to t2. These moments were below the laboratory reference values at t0 but above them at t1 and t2. Hip- and knee-rotation differences from the reference group persisted. **Conclusions:** Frontal-plane knee adaptations observed shortly before plate removal persisted, with small mean changes through skeletal maturity. Persistent kinetic and rotational differences suggest incomplete convergence toward the laboratory reference pattern. Further studies should examine the clinical relevance of these differences.

## 1. Introduction

Three-dimensional instrumented gait analysis characterizes lower-limb movements and internal joint moments throughout the gait cycle. By combining motion and ground-reaction force data, it identifies dynamic adaptations that may not be apparent from static alignment alone [1,2,3]. This is particularly relevant in idiopathic genu valgum, where gait deviations may persist despite correction of the coronal deformity.

This deformity has been associated with altered frontal-plane knee angles and moments and with coupled transverse-plane patterns, including reduced external knee rotation, increased external hip rotation, and changes in foot progression [4,5,6]. These variables are biomechanically interrelated: foot progression and trunk strategy can modify frontal-plane lever arms and joint moments during walking [7,8,9]. Correction of the coronal deformity may therefore be accompanied by changes in selected gait features while other kinematic patterns persist.

Transverse-plane rotational patterns may influence frontal-plane knee loading by altering the relationship between the knee and the ground-reaction-force vector during stance [6,10]. Higher frontal knee moments have been reported in limbs with persistent reduced external knee rotation after guided growth than in limbs with rotation within a published reference interval [11]. Whether persistent rotational patterns remain associated with differences in frontal knee moments after growth completion remains unclear.

Guided growth is an established approach for correcting angular deformities in skeletally immature patients [12]. Previous studies have described changes in frontal-plane knee kinematics and kinetics around implant removal, while transverse-plane deviations often persist [11,13,14]. Whether the frontal-plane changes persist during subsequent growth and into adulthood remains less clear.

The primary objective was to examine the longitudinal course of frontal-plane knee kinematics and internal knee moments, with particular emphasis on change from shortly before plate removal to final follow-up after skeletal maturity. Secondary and exploratory objectives were to characterize other gait variables, compare discrete outcomes and waveforms with laboratory reference data, and examine frontal knee moments in persistent knee-rotation phenotypes.

## 2. Materials and Methods

### 2.1. Study Design and Participants

This longitudinal gait-laboratory study followed patients treated for idiopathic genu valgum at the Orthopaedic Hospital Speising, Vienna, Austria. Gait assessments before guided growth (t0, 2014–2018) and shortly before plate removal (t1, 2016–2020) were retrieved retrospectively from the institutional database. Standardized final assessments after skeletal maturity (t2) were performed between 2022 and 2023. The same cohort contributed data at all three assessments.

Eligibility required tension-band plating for idiopathic genu valgum, analyzable three-dimensional gait recordings and corresponding standing long-leg radiographs, and a final assessment after 18 years of age. Exclusion criteria comprised neuromuscular disease, skeletal dysplasia, syndromic deformity, relevant limb-length discrepancy, failure to achieve correction, recurrent deformity requiring repeat guided growth, or major subsequent surgery of the same limb. Subsequent corrective procedures were excluded to examine the course following the initial treatment episode.

Of 87 patients identified, 35 were excluded during screening and 52 were invited for reassessment. Twenty-two attended, of whom 17 contributed complete, protocol-conforming data for 31 treated limbs. Fourteen participants contributed both treated limbs and three contributed one treated limb. The screening and technical exclusions are detailed in Supplementary Figure S1. The cohort size was determined by the available eligible participants with complete long-term assessments. The study was approved by the responsible ethics committee on 9 July 2020 (EK24/2020). Written informed consent was obtained before final assessment, and reporting followed the STROBE principles.

### 2.2. Guided Growth and Assessment Schedule

Treatment planning used standardized bilateral standing long-leg radiographs obtained in a two-leg stance with the knees centered. Mechanical axis deviation (MAD) established the extent of coronal malalignment. When correction was indicated, an abnormal mechanical lateral distal femoral angle (mLDFA) guided distal medial femoral plating, an abnormal mechanical medial proximal tibial angle (mMPTA) guided proximal medial tibial plating, and abnormalities at both levels guided combined plating. Open physes and sufficient remaining growth for correction were assessed before surgery. Hand radiographs for skeletal-age assessment were obtained selectively when remaining growth was uncertain.

Gait analysis was performed 4.39 ± 4.48 days before plate insertion (t0) and 5.74 ± 4.80 days before plate removal (t1). Routine follow-up was scheduled approximately every six months until growth completion. The final study assessment (t2) was scheduled after 18 years of age, with skeletal maturity confirmed by closed physes at the hip, knee, and distal tibia on long-leg radiographs.

The mechanical tibiofemoral angle (mTFA) was measured on the long-leg radiographs by two examiners. Their measurements were averaged for analysis and provided radiographic context for the gait findings. Positive values indicated valgus and negative values varus.

### 2.3. Three-Dimensional Gait Analysis

Gait data were collected barefoot at a self-selected speed along a 12-m walkway. Marker trajectories were recorded using a 17-camera system operating at 150 Hz (Vicon Motion Systems Ltd., Oxford, UK). Fifty passive reflective markers were placed using a combined model comprising a modified Cleveland Clinic lower-extremity component and the Plug-in Gait upper-body component [15]. Ground-reaction forces were recorded using three embedded force plates. One of two experienced, long-standing gait-laboratory staff members placed the markers using standardized anatomical landmarks and procedures. The same measurement system, force-plate configuration, marker definitions, and biomechanical model were used at all assessments.

Data preprocessing used Vicon Nexus Version 2.16 (Vicon Motion Systems Ltd., Oxford, UK) for trajectory reconstruction, marker labeling, gap filling, and filtering according to Woltring et al. [16]. Further processing used MATLAB, Version 2022b (The MathWorks, Inc., Natick, MA, USA). The processing workflow and software versions remained unchanged across all three assessments.

Walking trials were repeated until at least five valid force-plate contacts had been obtained for each side. Valid gait cycles were then averaged for analysis. Joint kinematics and internal moments were calculated from marker trajectories and force-plate data. Moments were normalized to body mass (Nm/kg), consistently with the laboratory protocol for the study and reference datasets. Negative transverse-plane values represented relative external rotation and positive values internal rotation. Negative frontal knee angles represented valgus. Positive internal frontal-plane knee moments represented internal valgus moments.

### 2.4. Outcome Measures

The primary outcomes were the mean frontal knee angle during single support and three internal frontal-plane knee-moment measures: the early-stance peak from initial contact to the end of mid-stance, the late-stance peak from terminal stance to toe-off, and the mean throughout stance. Their selection reflected the relevance of dynamic coronal alignment and stance-phase moment patterns to guided growth and previous gait studies [11,13,14]. These outcomes defined the biomechanical focus of this exploratory analysis.

Secondary outcomes comprised stance-phase knee and hip rotation, foot progression during single support, frontal hip angles and moments, frontal trunk lean, frontal knee range of motion, walking speed, normalized walking speed, cadence, normalized cadence, and step width. Frontal knee range of motion was the maximum minus minimum angle over the complete gait cycle. Hip mechanics were quantified by early- and late-stance maxima of the frontal angle and internal frontal moment. Trunk lean was quantified by the stance minimum and whole-cycle mean. Marker-derived hip and knee rotations describe the relative orientation of adjacent body segments during gait and do not directly measure anatomical femoral or tibial torsion.

Walking speed and cadence were normalized using leg length (L) as the body-size scaling variable. This anthropometric scaling reduces the influence of body dimensions and growth on comparisons between individuals and assessments [17,18]. Residual associations with age and body size may remain after normalization [19]. Joint moments retained the separate mass-based normalization described above.

The exploratory phenotype analysis used the mean ±1 standard deviation of the typically developing group reported by Farr et al. [11] to classify knee rotation at t1 and t2. Categories were rotation within this published reference interval and reduced external knee rotation. The threshold provided consistency with the earlier classification and was not a validated cutoff for torsional pathology. Only limbs with the same category at t1 and t2 were included, focusing the comparison on persistent dynamic patterns.

### 2.5. Laboratory Reference Data

The laboratory reference group comprised 11 healthy participants (22 limbs). Mean ± standard deviation values were 12.91 ± 4.55 years for age (range, 8–20 years), 154.0 ± 16.3 cm for height, and 47.2 ± 16.6 kg for body mass. Data were acquired in the same laboratory using the same protocol. This group provided a common reference for all three assessments, as separate age-stratified datasets were not available. The comparisons were exploratory because the reference group was not matched by age and sex to each assessment. Spatiotemporal comparisons used participant-level observations, and kinematic and kinetic comparisons used limb-level observations.

Waveform comparisons included frontal hip and knee angles, internal frontal hip and knee moments, hip and knee rotation, foot progression, and frontal trunk lean over the time-normalized gait cycle. The figures display mean waveforms with ±1 SD. Discrete outcomes quantify scalar features within specified phases or across the cycle, whereas waveform tests assess time-dependent differences. Because these analyses assess different features of gait, their statistical findings may differ.

### 2.6. Statistical Analysis

Continuous variables are presented as mean ± standard deviation and categorical variables as absolute numbers (n). Longitudinal discrete gait outcomes were analyzed using linear mixed-effects models (LMMs), with time as a categorical fixed effect. Limb-level models included random intercepts for participant and limb nested within participant, accounting for dependence between both limbs and repeated measurements of the same limb. Spatiotemporal variables contributed one observation per participant and assessment and were analyzed with a repeated-measures mixed model at participant level. Analyses used complete cases across all three assessments: 31 limbs from 17 participants for limb-level outcomes and 17 participants for spatiotemporal outcomes.

Models were fitted by restricted maximum likelihood in Python (version 3.14.6) using NumPy (version 2.5.1) and SciPy (version 1.18.0). Three pairwise time contrasts were evaluated for each outcome: t1−t0, t2−t0, and t2−t1. Model-estimated changes are reported in the original units with 95% confidence intervals (CIs). Tests and CIs used a t distribution with 16 degrees of freedom, approximated as the number of participants minus one. Pairwise *p* values were Bonferroni-adjusted across the three contrasts within each outcome, whereas CIs were unadjusted. No correction was applied across outcomes, and the results were interpreted as exploratory.

Additional repeated-measures ANOVA (rmANOVA) analyses provided a comparison with the LMM results. Normality was assessed with the Shapiro–Wilk test and sphericity with Mauchly’s test, with Greenhouse–Geisser or Huynh–Feldt corrections when required. Pairwise comparisons were Bonferroni-adjusted within each outcome, and Cohen’s f quantified the overall time effect for the four primary outcomes [20]. Longitudinal interpretation was based on the LMMs.

Comparisons with the laboratory reference group used two-sided Welch tests with mean differences and 95% CIs. Phenotype comparisons used independent-samples *t*-tests, and the t1-to-t2 mTFA comparison used a paired *t*-test.

Waveform comparisons used one-dimensional statistical parametric mapping (SPM) [21]. Longitudinal SPM rmANOVA tested the overall time effect across t0, t1, and t2, followed by pairwise comparisons. Separate SPM tests compared the cohort with the laboratory reference group at each assessment. The additional rmANOVA, reference-group, phenotype, radiographic, and waveform analyses were exploratory. For limb-level outcomes, these additional analyses did not account for dependence between both limbs of a participant. All *t*-tests were two-sided, with *p* ≤ 0.05 as the significance threshold.

## 3. Results

### 3.1. Cohort Characteristics

The cohort included 11 male and 6 female participants. Mean age was 13.53 ± 1.27 years at t0, 14.67 ± 1.45 years at t1, and 19.64 ± 1.08 years at t2. Seventeen limbs underwent isolated distal femoral plating, 9 isolated proximal tibial plating, and 5 combined femoral and tibial plating. Mean follow-up from plate removal to t2 was 59.66 ± 12.13 months (Table 1).

The mTFA changed from 5.03° ± 1.91° valgus at t0 to −0.10° ± 2.47° at t1 and 1.68° ± 2.41° valgus at t2. The t1-to-t2 comparison indicated a 1.78° shift toward valgus (paired *t*-test, t(30) = 4.26, *p* < 0.001). These radiographic values provide context for the gait findings.

### 3.2. Primary Frontal-Plane Knee Outcomes

**Figure 1:**
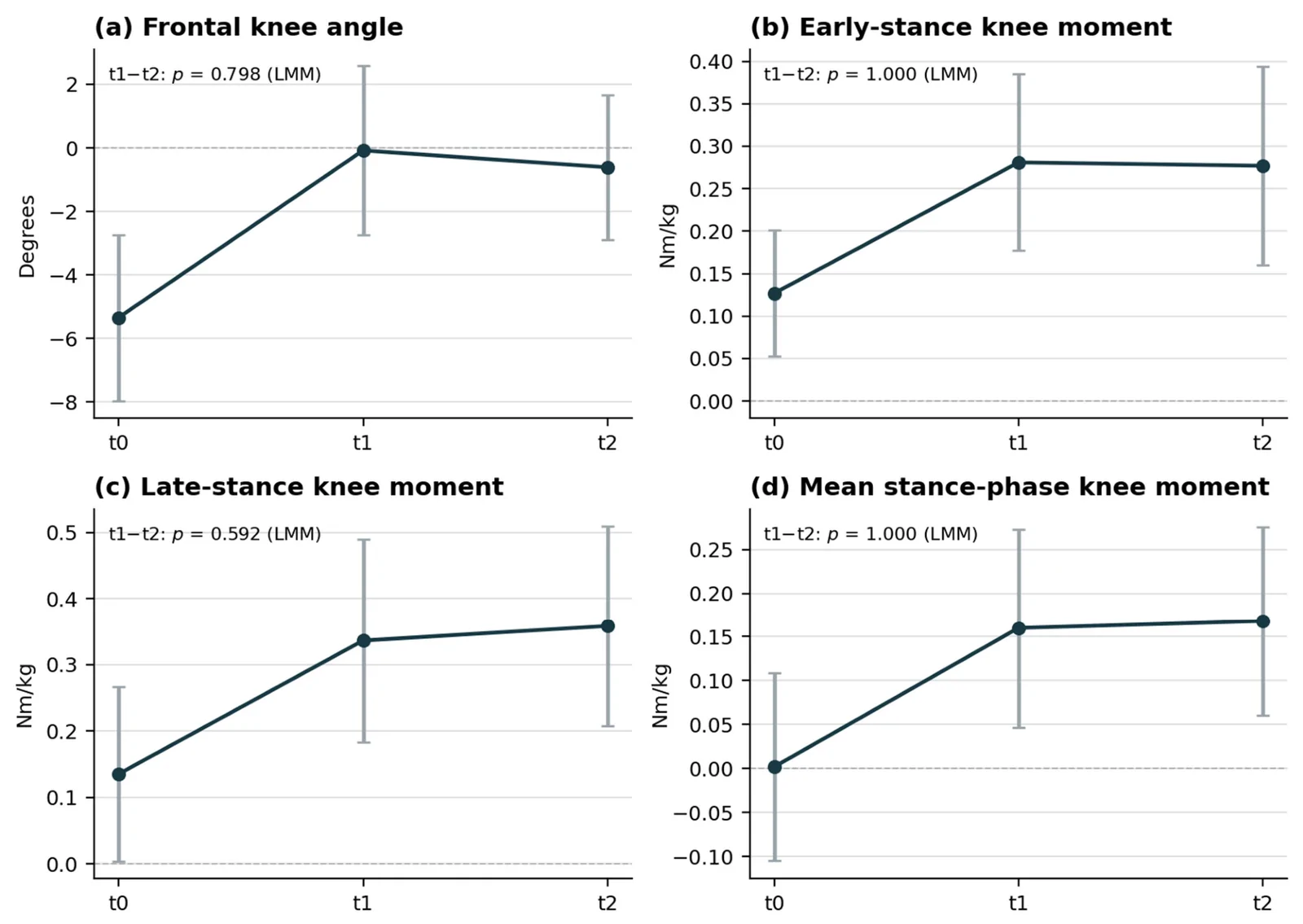
Longitudinal course of the primary gait outcomes at preoperative assessment (t0), shortly before plate removal (t1), and final follow-up after skeletal maturity (t2). Points and bars represent means and ±1 SD. Displayed *p* values are from the t1-to-t2 LMM contrasts, Bonferroni-adjusted within each outcome.

The estimated frontal knee-angle change from t0 to t1 was +5.26° (95% CI, 4.29° to 6.23°; *p* < 0.001), indicating less dynamic valgus. All three internal frontal-plane knee-moment measures increased over this interval (all *p* < 0.001). From t1 to t2, the estimated changes were −0.53° for the knee angle (95% CI, −1.50° to 0.44°; *p* = 0.798), −0.004 Nm/kg for the early-stance moment (−0.035 to 0.027; *p* = 1.000), +0.022 Nm/kg for the late-stance moment (−0.013 to 0.057; *p* = 0.592), and +0.008 Nm/kg for the stance mean (−0.018 to 0.034; *p* = 1.000). Estimated mean changes after t1 were small (Table 2; Figure 1).

### 3.3. Secondary Gait Outcomes

**Figure 2:**
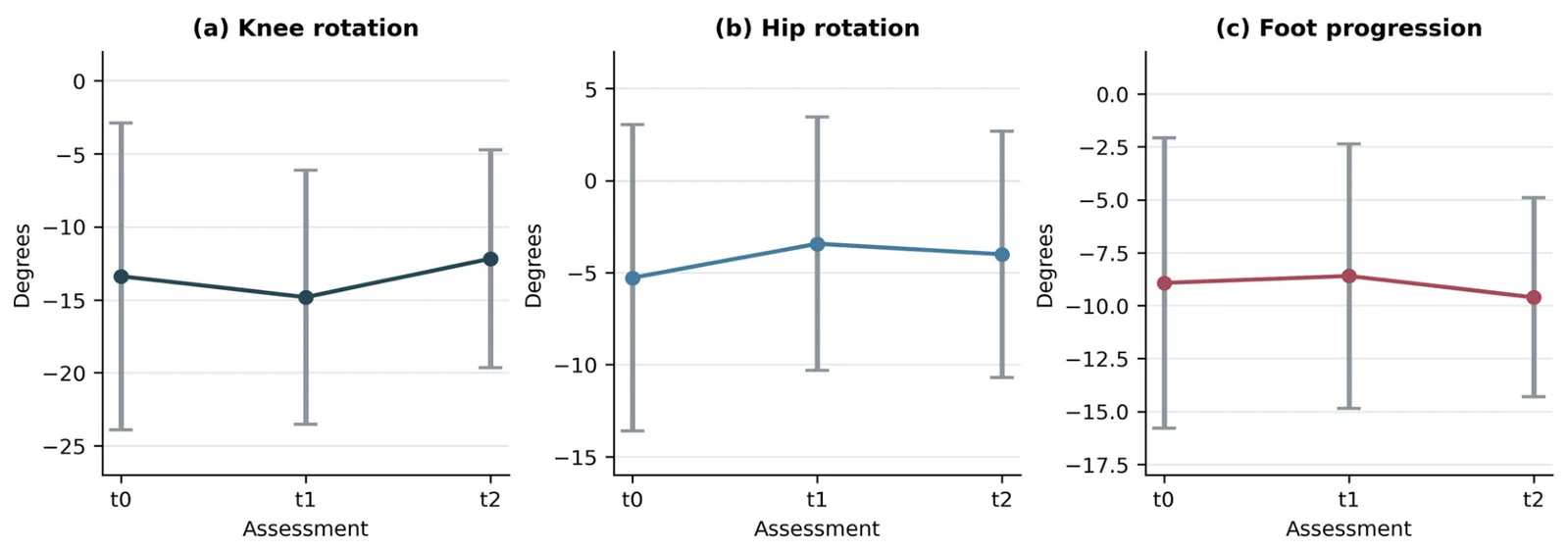
Longitudinal course of (**a**) knee rotation, (**b**) hip rotation, and (**c**) foot progression at t0, shortly before plate removal (t1), and final follow-up after skeletal maturity (t2). Points and bars represent means and ±1 SD. Negative values indicate relative external rotation. No significant t1-to-t2 change was detected by the LMMs.

Walking speed and normalized walking speed did not differ significantly across assessments. Cadence was lower at t2 than at t0 (*p* = 0.009), while normalized cadence showed no significant change. Step width decreased from t0 to t1 (*p* = 0.003). No significant t1-to-t2 changes were detected for foot progression, hip or knee rotation, frontal knee range of motion, or trunk lean (Table 3; Figure 2).

Both early- and late-stance frontal hip angles decreased from t0 to t1 (*p* = 0.021 and *p* = 0.002). The late-stance angle increased from 3.85° ± 2.88° at t1 to 6.13° ± 3.26° at t2 (*p* = 0.002), while neither angle showed a significant t0-to-t2 difference. The early-stance internal frontal hip moment increased from t0 to t1 (*p* = 0.025), whereas the late-stance moment increased from 0.69 ± 0.17 to 0.80 ± 0.17 Nm/kg between t1 and t2 (*p* < 0.001). Both moments were higher at t2 than at t0 (both *p* < 0.001). Secondary LMM change estimates and CIs are provided in Supplementary Table S1. Additional rmANOVA results are presented in Supplementary Table S2, and longitudinal waveform comparisons in Supplementary Figure S2.

### 3.4. Comparisons with the Laboratory Reference Group

At t0, the study cohort had a more valgus frontal knee angle, more external hip rotation, less external knee rotation, and lower internal frontal-plane knee moments than the laboratory reference group. The knee-angle difference was −4.83° (95% CI, −6.26° to −3.41°). At t1 and t2, the corresponding differences were +0.43° (−1.01° to +1.86°) and −0.10° (−1.46° to +1.25°), respectively. All three knee-moment measures were higher than the reference values at t1 and t2. At t2, the differences were +0.063 Nm/kg (95% CI, +0.004 to +0.122) for the early-stance peak, +0.106 Nm/kg (+0.029 to +0.183) for the late-stance peak, and +0.067 Nm/kg (+0.013 to +0.121) for the stance mean. Discrete hip- and knee-rotation differences were detected at all assessments, whereas foot progression did not differ significantly from the reference group. Walking speed and normalized walking speed did not differ significantly from the reference values (Table 4).

**Figure 3:**
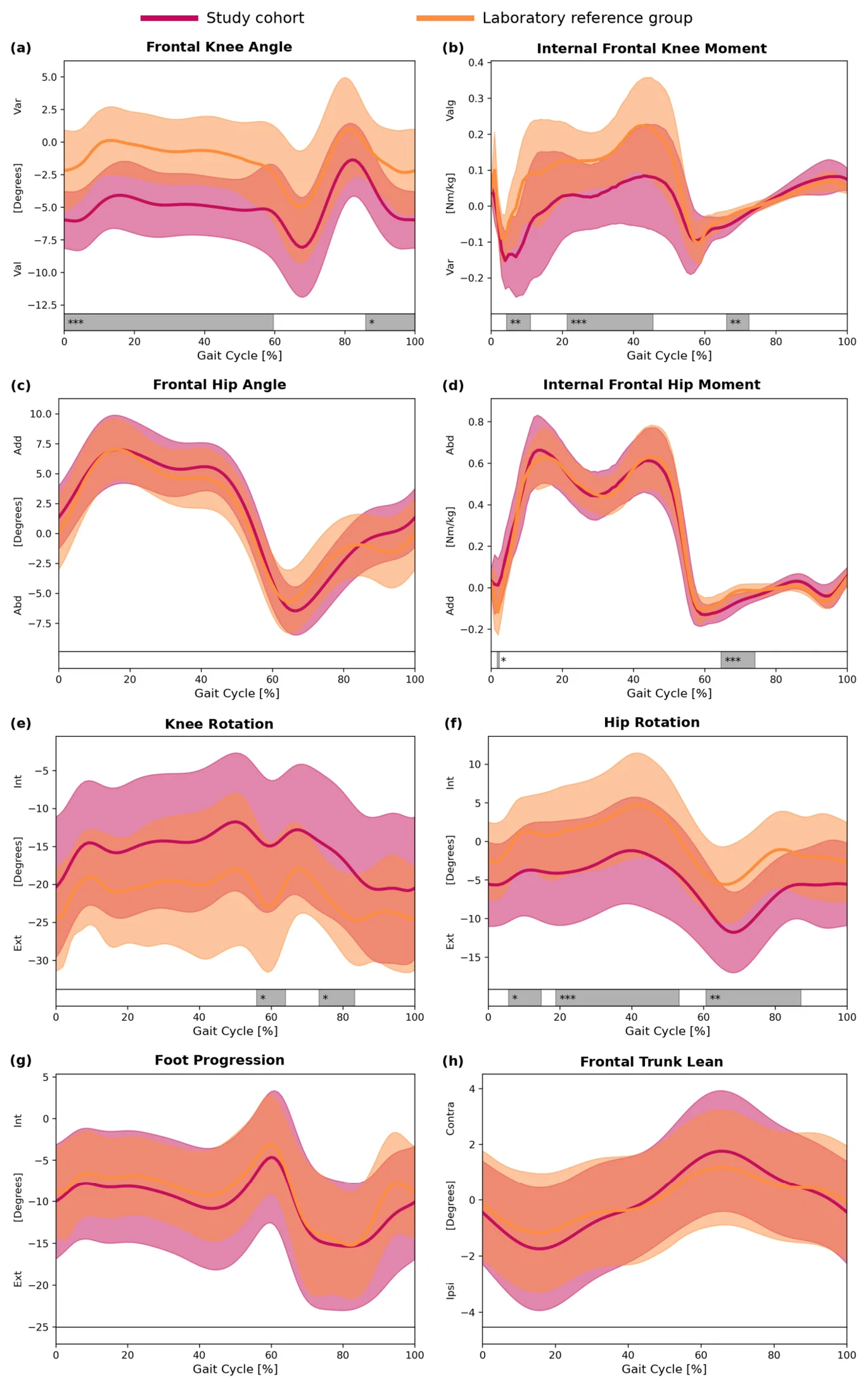
Time-normalized gait-cycle waveforms for the study cohort and the laboratory reference group at preoperative assessment (t0): (**a**) frontal knee angle; (**b**) internal frontal knee moment; (**c**) frontal hip angle; (**d**) internal frontal hip moment; (**e**) knee rotation; (**f**) hip rotation; (**g**) foot progression; and (**h**) frontal trunk lean. Magenta lines represent the study cohort and orange lines the laboratory reference group; shaded bands represent mean ±1 SD. Grey bars identify gait-cycle regions with statistically significant between-group differences in SPM. * *p* ≤ 0.05; ** *p* ≤ 0.01; *** *p* ≤ 0.001.

**Figure 4:**
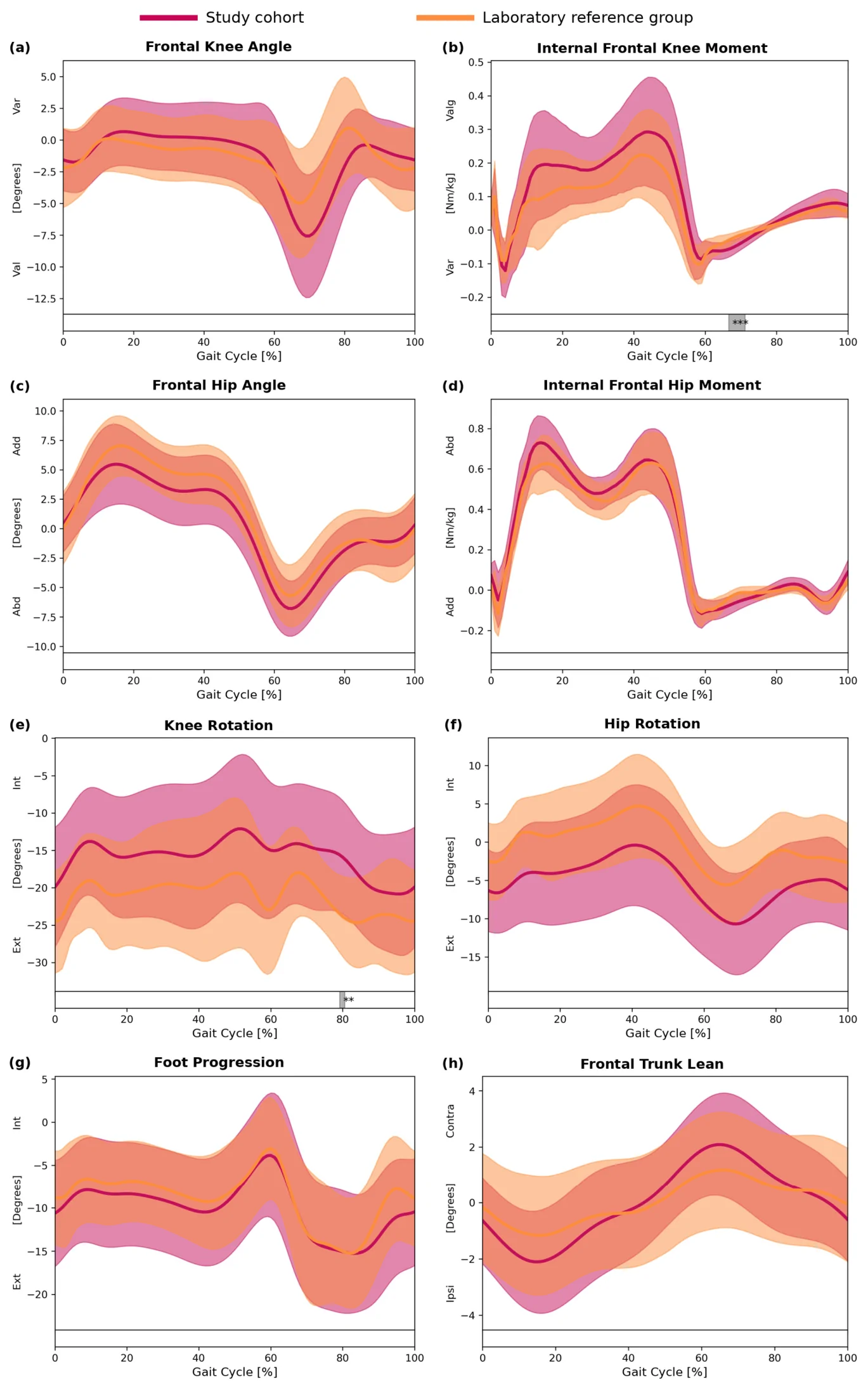
Time-normalized gait-cycle waveforms for the study cohort and the laboratory reference group at the assessment shortly before plate removal (t1): (**a**) frontal knee angle; (**b**) internal frontal knee moment; (**c**) frontal hip angle; (**d**) internal frontal hip moment; (**e**) knee rotation; (**f**) hip rotation; (**g**) foot progression; and (**h**) frontal trunk lean. Magenta lines represent the study cohort and orange lines the laboratory reference group; shaded bands represent mean ±1 SD. Grey bars identify gait-cycle regions with statistically significant between-group differences in SPM. ** *p* ≤ 0.01; *** *p* ≤ 0.001.

**Figure 5:**
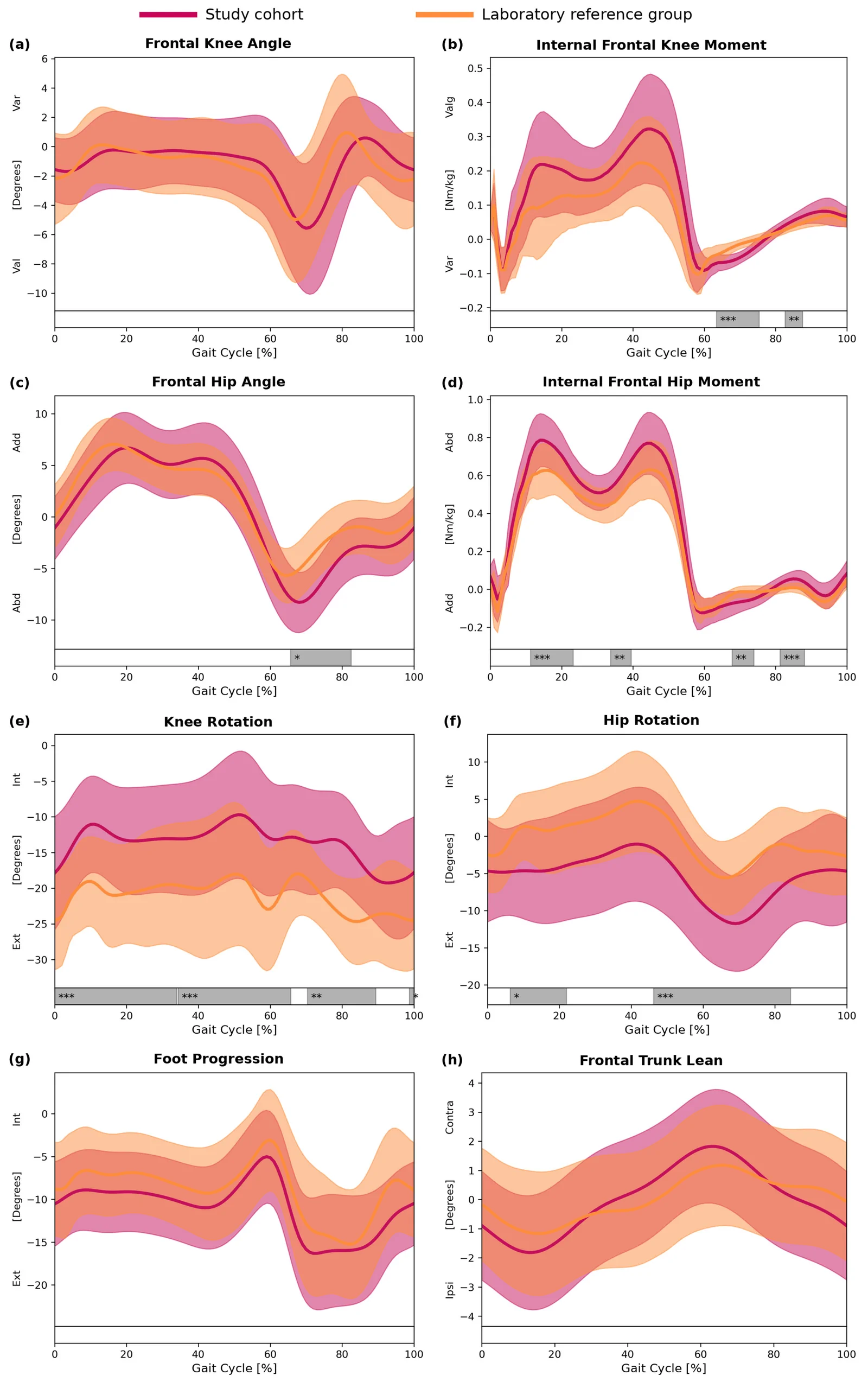
Time-normalized gait-cycle waveforms for the study cohort and the laboratory reference group at final follow-up after skeletal maturity (t2): (**a**) frontal knee angle; (**b**) internal frontal knee moment; (**c**) frontal hip angle; (**d**) internal frontal hip moment; (**e**) knee rotation; (**f**) hip rotation; (**g**) foot progression; and (**h**) frontal trunk lean. Magenta lines represent the study cohort and orange lines the laboratory reference group; shaded bands represent mean ±1 SD. Grey bars identify gait-cycle regions with statistically significant between-group differences in SPM. * *p* ≤ 0.05; ** *p* ≤ 0.01; *** *p* ≤ 0.001.

Waveform comparisons provided phase-specific information. At t0, significant between-group gait-cycle regions were identified for the frontal knee angle, internal frontal knee and hip moments, and hip and knee rotation. At t1, significant regions were limited to brief portions of the internal frontal knee-moment and knee-rotation waveforms. At t2, significant regions involved the internal frontal knee and hip moments, hip and knee rotation, and a limited portion of the frontal hip-angle waveform; no significant region was identified for the frontal knee angle. Foot progression and frontal trunk lean showed no significant regions at any assessment (Figure 3, Figure 4 and Figure 5).

### 3.5. Exploratory Knee-Rotation Phenotype

Twenty-one limbs had the same knee-rotation classification at t1 and t2: 13 remained within the published reference interval and eight showed reduced external rotation. Ten limbs did not meet this persistent-category criterion. Mean frontal knee moments were numerically higher in the reduced-external-rotation group at t1 and t2, particularly for the stance mean, but no exploratory between-group comparison was statistically significant. Both groups are presented in Table 5.

## 4. Discussion

The principal finding was the persistence of the frontal-plane knee adaptations present shortly before plate removal over approximately five years to skeletal maturity. LMMs accounting for participant and limb dependence showed marked preoperative-to-t1 changes and comparatively small estimated t1-to-t2 changes. These findings extend the assessment of guided growth beyond the initial correction, while also documenting persistent kinetic and rotational differences from the laboratory reference group.

The preoperative combination of dynamic valgus, altered rotation, and lower internal frontal knee moments is consistent with earlier descriptions of idiopathic genu valgum [4]. Studies by Stevens et al., Böhm et al., and Farr et al. also documented frontal-plane changes around plate removal, with residual dynamic deviations in some limbs [11,13,14]. A shorter study using wearable sensors in a mixed valgus/varus cohort found no significant change in most spatiotemporal measures [22]. The present study extends instrumented follow-up into early adulthood and focuses on the course after the initial correction.

The mean mTFA shifted 1.78° toward valgus after plate removal. This radiographic shift occurred alongside comparatively small estimated changes in the primary gait outcomes. These group-level observations illustrate the complementary information provided by static and dynamic assessment, consistent with modeling evidence that coronal alignment and dynamic loading are not interchangeable [23]. Whether individual radiographic changes were associated with changes in gait was not examined.

The broad preoperative frontal knee-angle difference in the waveform comparison was no longer detected at t1 or t2, complementing the longitudinal findings.

All three internal frontal-plane knee-moment measures shifted from below the laboratory reference values at t0 to above them at t1 and t2. The direction of this sustained kinetic change cannot automatically be interpreted as biomechanical improvement. Modeling and experimental studies link joint moments to compartment loading [24,25], but internal moments remain indirect measures and do not directly quantify compartment-specific contact forces [26]. The clinical relevance of these persistent kinetic differences warrants further investigation.

No significant longitudinal changes were detected for hip rotation, knee rotation, or foot progression. At t2, hip rotation was more externally directed and knee rotation less externally directed than in the laboratory reference group, while foot progression showed no significant difference. Significant SPM regions for hip and knee rotation were present at t0 and t2, whereas at t1 only brief knee-rotation regions were detected. These time-specific waveform findings complement the discrete comparisons, which evaluate phase summaries rather than the full time course. Persistent transverse-plane patterns after coronal correction have also been reported by Farr et al. [11].

Rotational alignment, foot progression, and trunk strategy can influence frontal-plane loading [5,6,7,8,10]. Radler et al. reported an association between gait-derived knee rotation and CT-based tibial torsion in patients with established rotational malalignment [27]. In the present cohort, gait-derived rotation describes dynamic segment orientation and should be interpreted alongside clinical examination and imaging when further treatment is considered.

Farr et al. reported higher frontal knee moments in limbs with persistent reduced external knee rotation after guided growth [11], and preliminary modeling findings suggest that rotational alignment may influence contact-force distribution despite coronal correction [28]. The corresponding moment means were also numerically higher in our reduced-external-rotation group, although none of the comparisons between the 13 reference-interval and eight reduced-external-rotation limbs reached statistical significance. This pattern merits examination in larger cohorts using knee rotation as a continuous measure.

The hip variables followed different temporal courses: frontal angles initially decreased and approached their preoperative means at t2, while increases in early- and late-stance moments occurred over different intervals. These secondary changes may reflect growth, maturation, or altered gait strategy. Walking speed also affects pediatric gait mechanics [29,30]. Although mean speed changed little, residual speed effects cannot be excluded. The decrease in raw cadence without a significant change in normalized cadence is compatible with growth-related adaptation. Age-related kinetic changes [31] should also be considered when comparing these findings with the younger laboratory reference group.

Clinically, the study documents the long-term course of measured gait adaptations after coronal correction. Instrumented gait analysis may add information when symptoms, persistent rotational patterns, or discrepancies between static and dynamic findings prompt further assessment. Future prospective studies combining gait analysis with pain, physical function, activity, and sports participation would help establish the clinical relevance of these biomechanical findings.

Several limitations should be considered. Only 17 of 52 invited patients contributed complete data. Baseline characteristics were not compared between participants and nonparticipants, and detailed reasons for nonparticipation and recurrence-to-reoperation intervals of excluded patients were not evaluated. Excluding repeat guided growth and major subsequent surgery allowed assessment of the initial treatment course, but participation and treatment-related exclusions may have favored patients with successful correction.

The LMMs accounted for participant and limb dependence in longitudinal discrete gait outcomes. Precision was limited by the 17 independent participants, and tests and CIs relied on an approximate degrees-of-freedom rule. The additional rmANOVA, reference-group, phenotype, radiographic, and waveform analyses did not account for dependence between both limbs of a participant. Their standard errors may therefore be underestimated, increasing the risk of false-positive results. Multiple comparisons across outcomes add to this risk. The results should therefore be interpreted as exploratory. Nonsignificant findings do not establish equivalence or exclude clinically relevant differences.

The correction site was selected according to the anatomical source of valgus. Whether femoral, tibial, and combined correction were associated with different gait responses could not be assessed reliably in this cohort. Restricting the phenotype analysis to the 21 limbs with the same classification at t1 and t2 may also have selected particular rotational patterns.

Marker-derived transverse-plane kinematics are sensitive to marker placement and soft-tissue artifact despite standardized procedures and experienced assessors. Comparable clinical rotational profiles and anatomical torsion measurements were not available across assessments, limiting attribution of the dynamic patterns to a specific anatomical cause. Interobserver reliability of the radiographic measurements was not quantified.

The laboratory reference group was small and was not matched by age and sex to each assessment. Without an untreated genu valgum group, treatment-related changes could not be separated from normal maturation. Mass normalization of moments may leave residual associations with body size and age [19]. Patient-reported pain, function, activity, satisfaction, and sports participation were not assessed longitudinally.

## 5. Conclusions

In this selected cohort, frontal-plane knee adaptations present shortly before plate removal showed small mean changes over approximately five years to skeletal maturity. Hip- and knee-rotation differences and higher internal frontal knee moments relative to the laboratory reference group remained. Together, these findings support the persistence of selected gait adaptations after coronal correction. Future studies linking gait patterns to clinical outcomes would help establish their clinical relevance.

## Figures and Tables

**Table 1:** Demographic and treatment characteristics.

Characteristic	Value
Participants, *n*	17
Treated limbs, *n*	31
Male/female participants, *n*	11/6
Participants contributing both/one treated limb, n	14/3
Age at t0, years	13.53 ± 1.27
Age at t1, years	14.67 ± 1.45
Age at t2, years	19.64 ± 1.08
Isolated femoral/isolated tibial/combined plating, limbs	17/9/5
Implant duration, months	13.66 ± 8.58
Follow-up from plate removal to t2, months	59.66 ± 12.13

Continuous values are presented as mean ± standard deviation.

**Table 2:** Primary frontal-plane knee outcomes and longitudinal mixed-model contrasts.

Outcome	t0	t1	t2	t1 − t0 Δ (95% CI) *p* Value	t2 − t1 Δ (95% CI) *p* Value	t2 − t0 Δ (95% CI) *p* Value
Frontal knee angle, °	−5.34 ± 2.61	−0.08 ± 2.66	−0.61 ± 2.27	+5.26 (4.29 to 6.23) **<0.001**	−0.53 (−1.50 to 0.44) 0.798	+4.73 (3.76 to 5.70) **<0.001**
Internal frontal knee moment, early maximum, Nm/kg	0.127 ± 0.074	0.281 ± 0.104	0.277 ± 0.117	+0.154 (0.123 to 0.185) **<0.001**	−0.004 (−0.035 to 0.027) 1.000	+0.150 (0.119 to 0.181) **<0.001**
Internal frontal knee moment, late maximum, Nm/kg	0.135 ± 0.131	0.336 ± 0.153	0.358 ± 0.151	+0.201 (0.166 to 0.236) **<0.001**	+0.022 (−0.013 to 0.057) 0.592	+0.223 (0.188 to 0.258) **<0.001**
Internal frontal knee moment, stance mean, Nm/kg	0.002 ± 0.107	0.160 ± 0.113	0.168 ± 0.108	+0.158 (0.132 to 0.184) **<0.001**	+0.008 (−0.018 to 0.034) 1.000	+0.166 (0.140 to 0.192) **<0.001**

Time-point values are mean ± standard deviation. Δ denotes the LMM estimate for the later minus earlier assessment, with its nominal 95% CI. Pairwise *p* values are Bonferroni-adjusted across three time contrasts within each outcome. Models included 31 limbs from 17 participants. Negative frontal knee angles indicate valgus; positive internal frontal knee moments indicate valgus moments. Bold *p* values indicate *p* ≤ 0.05.

**Table 3:** Exploratory secondary gait outcomes and Bonferroni-adjusted mixed-model comparisons.

Outcome	t0	t1	t2	*p* t0–t1	*p* t1–t2	*p* t0–t2
Walking speed, m/s	1.26 ± 0.09	1.28 ± 0.10	1.29 ± 0.10	1.000	1.000	1.000
Normalized walking speed	0.44 ± 0.03	0.44 ± 0.03	0.43 ± 0.04	1.000	1.000	1.000
Cadence, steps/min	114.27 ± 8.45	111.17 ± 5.25	109.15 ± 5.91	0.149	0.556	**0.009**
Normalized cadence	0.56 ± 0.04	0.55 ± 0.03	0.55 ± 0.02	0.510	1.000	0.510
Step width, m	0.10 ± 0.02	0.08 ± 0.02	0.09 ± 0.02	**0.003**	0.177	0.177
Foot progression, °	−8.93 ± 6.86	−8.60 ± 6.25	−9.61 ± 4.70	1.000	0.559	1.000
Hip rotation, °	−5.29 ± 8.33	−3.44 ± 6.89	−4.01 ± 6.69	0.218	1.000	0.606
Knee rotation, °	−13.39 ± 10.51	−14.82 ± 8.69	−12.19 ± 7.46	0.812	0.156	1.000
Frontal hip angle, early maximum, °	7.28 ± 2.92	5.71 ± 3.45	6.92 ± 3.20	**0.021**	0.089	1.000
Frontal hip angle, late maximum, °	6.25 ± 2.22	3.85 ± 2.88	6.13 ± 3.26	**0.002**	**0.002**	1.000
Internal frontal hip moment, early maximum, Nm/kg	0.72 ± 0.15	0.78 ± 0.13	0.82 ± 0.13	**0.025**	0.186	**<0.001**
Internal frontal hip moment, late maximum, Nm/kg	0.65 ± 0.15	0.69 ± 0.17	0.80 ± 0.17	0.281	**<0.001**	**<0.001**
Frontal knee range of motion, °	10.73 ± 3.73	11.47 ± 4.24	10.20 ± 3.64	0.794	0.194	1.000
Trunk lean, stance minimum, °	−2.31 ± 2.08	−2.53 ± 1.82	−2.19 ± 1.99	1.000	0.881	1.000
Trunk lean, cycle mean, °	−0.07 ± 1.69	−0.02 ± 1.42	0.07 ± 1.68	1.000	1.000	1.000

Values are mean ± standard deviation. LMM *p* values are Bonferroni-adjusted across three time contrasts within each outcome. Spatiotemporal outcomes use 17 participants; kinematic and kinetic outcomes use 31 limbs from these participants. Frontal knee range of motion refers to the complete gait cycle; early and late hip maxima refer to the corresponding stance phases. Negative transverse-plane values indicate relative external rotation. Bold *p* values indicate *p* ≤ 0.05.

**Table 4:** Study-cohort values and exploratory comparisons with the laboratory reference group.

Outcome	Laboratory Reference	t0	t1	t2
Cadence, steps/min	117.58 ± 8.32	114.27 ± 8.45 Δ −3.31 (95% CI −10.03 to +3.42) *p* = 0.319	**111.17 ± 5.25** **Δ −6.41 (95% CI −12.40 to −0.42)** *p* = 0.038 *	**109.15 ± 5.91** **Δ −8.43 (95% CI −14.54 to −2.32)** *p* = 0.010 *
Step width, m	0.075 ± 0.018	**0.10 ± 0.02** **Δ +0.025 (95% CI +0.010 to +0.040)** *p* = 0.002 *	0.08 ± 0.02 Δ +0.005 (95% CI −0.010 to +0.020) *p* = 0.486	**0.09 ± 0.02** **Δ +0.015 (95% CI +0.000 to +0.030)** *p* = 0.047 *
Walking speed, m/s	1.223 ± 0.118	1.26 ± 0.09 Δ +0.037 (95% CI −0.051 to +0.125) *p* = 0.388	1.28 ± 0.10 Δ +0.057 (95% CI −0.033 to +0.147) *p* = 0.202	1.29 ± 0.10 Δ +0.067 (95% CI −0.023 to +0.157) *p* = 0.136
Normalized walking speed	0.454 ± 0.035	0.44 ± 0.03 Δ −0.014 (95% CI −0.041 to +0.013) *p* = 0.288	0.44 ± 0.03 Δ −0.014 (95% CI −0.041 to +0.013) *p* = 0.288	0.43 ± 0.04 Δ −0.024 (95% CI −0.054 to +0.006) *p* = 0.107
Foot progression, °	−7.80 ± 4.90	−8.93 ± 6.86 Δ −1.13 (95% CI −4.38 to +2.11) *p* = 0.486	−8.60 ± 6.25 Δ −0.80 (95% CI −3.88 to +2.28) *p* = 0.603	−9.61 ± 4.70 Δ −1.81 (95% CI −4.52 to +0.89) *p* = 0.184
Hip rotation, °	1.48 ± 5.44	**−5.29 ± 8.33** **Δ −6.77 (95% CI −10.57 to −2.97)** *p* < 0.001 *	**−3.44 ± 6.89** **Δ −4.92 (95% CI −8.33 to −1.51)** *p* = 0.005 *	**−4.01 ± 6.69** **Δ −5.49 (95% CI −8.84 to −2.14)** *p* = 0.002 *
Internal frontal hip moment, early maximum, Nm/kg	0.681 ± 0.121	0.72 ± 0.15 Δ +0.039 (95% CI −0.036 to +0.114) *p* = 0.301	**0.78 ± 0.13** **Δ +0.099 (95% CI +0.029 to +0.169)** *p* = 0.007 *	**0.82 ± 0.13** **Δ +0.139 (95% CI +0.069 to +0.209)** *p* < 0.001 *
Internal frontal hip moment, late maximum, Nm/kg	0.668 ± 0.148	0.65 ± 0.15 Δ −0.018 (95% CI −0.102 to +0.065) *p* = 0.662	0.69 ± 0.17 Δ +0.022 (95% CI −0.066 to +0.110) *p* = 0.622	**0.80 ± 0.17** **Δ +0.132 (95% CI +0.044 to +0.220)** *p* = 0.004 *
Frontal knee angle, °	−0.51 ± 2.49	**−5.34 ± 2.61** **Δ −4.83 (95% CI −6.26 to −3.41)** *p* < 0.001 *	−0.08 ± 2.66 Δ +0.43 (95% CI −1.01 to +1.86) *p* = 0.555	−0.61 ± 2.27 Δ −0.10 (95% CI −1.46 to +1.25) *p* = 0.876
Knee rotation, °	−20.15 ± 7.84	**−13.39 ± 10.51** **Δ +6.76 (95% CI +1.70 to +11.82)** *p* = 0.010 *	**−14.82 ± 8.69** **Δ +5.33 (95% CI +0.73 to +9.93)** *p* = 0.024 *	**−12.19 ± 7.46** **Δ +7.96 (95% CI +3.64 to +12.27)** *p* < 0.001 *
Knee moment, early maximum, Nm/kg	0.214 ± 0.096	**0.127 ± 0.074** **Δ −0.087 (95% CI −0.137 to −0.038)** *p* < 0.001 *	**0.281 ± 0.104** **Δ +0.067 (95% CI +0.011 to +0.122)** *p* = 0.020 *	**0.277 ± 0.117** **Δ +0.063 (95% CI +0.004 to +0.122)** *p* = 0.038 *
Knee moment, late maximum, Nm/kg	0.252 ± 0.127	**0.135 ± 0.131** **Δ −0.117 (95% CI −0.189 to −0.045)** *p* = 0.002 *	**0.336 ± 0.153** **Δ +0.084 (95% CI +0.006 to +0.161)** *p* = 0.034 *	**0.358 ± 0.151** **Δ +0.106 (95% CI +0.029 to +0.183)** *p* = 0.008 *
Knee moment, stance mean, Nm/kg	0.101 ± 0.087	**0.002 ± 0.107** **Δ −0.099 (95% CI −0.152 to −0.045)** *p* < 0.001 *	**0.160 ± 0.113** **Δ +0.059 (95% CI +0.004 to +0.115)** *p* = 0.036 *	**0.168 ± 0.108** **Δ +0.067 (95% CI +0.013 to +0.121)** *p* = 0.015 *
Trunk lean, stance minimum, °	−1.53 ± 2.06	−2.31 ± 2.08 Δ −0.78 (95% CI −1.94 to +0.38) *p* = 0.182	−2.53 ± 1.82 Δ −1.00 (95% CI −2.11 to +0.10) *p* = 0.074	−2.19 ± 1.99 Δ −0.66 (95% CI −1.80 to +0.48) *p* = 0.248
Trunk lean, cycle mean, °	0.02 ± 1.90	−0.07 ± 1.69 Δ −0.09 (95% CI −1.11 to +0.93) *p* = 0.865	−0.02 ± 1.42 Δ −0.04 (95% CI −1.01 to +0.93) *p* = 0.939	0.07 ± 1.68 Δ +0.05 (95% CI −0.96 to +1.07) *p* = 0.916

Study and laboratory-reference values are mean ± standard deviation. For each assessment, the study values are followed by the difference from the reference group (Δ = study minus reference), its 95% confidence interval, and the two-sided Welch *p* value. Participant-level comparisons included 17 study and 11 reference participants; limb-level comparisons included 31 and 22 limbs, respectively. Negative transverse-plane values indicate relative external rotation. Statistically significant values are in bold. * *p* ≤ 0.05.

**Table 5:** Frontal knee moments according to persistent knee-rotation phenotype.

Knee-Moment Outcome	Time	Reference-Interval Phenotype (*n* = 13)	Reduced External Rotation (*n* = 8)	*p* Value
Early maximum, Nm/kg	t0	0.142 ± 0.076	0.115 ± 0.087	0.468
	t1	0.260 ± 0.123	0.333 ± 0.085	0.156
	t2	0.256 ± 0.127	0.326 ± 0.131	0.237
Late maximum, Nm/kg	t0	0.137 ± 0.177	0.143 ± 0.010	0.935
	t1	0.297 ± 0.207	0.408 ± 0.100	0.175
	t2	0.314 ± 0.191	0.435 ± 0.119	0.126
Stance mean, Nm/kg	t0	−0.011 ± 0.139	0.014 ± 0.094	0.666
	t1	0.125 ± 0.068	0.229 ± 0.068	0.070
	t2	0.129 ± 0.133	0.226 ± 0.091	0.087

Values are mean ± standard deviation. Phenotypes follow the published classification of Farr et al. [11]. Only limbs with the same classification at t1 and t2 are included. The *p* values are from exploratory independent-samples *t*-tests.

## Data Availability

The original contributions presented in the study are included in the article material, further inquiries can be directed to the corresponding author.

## Supplementary Materials

The following supporting information can be downloaded at: https://www.mdpi.com/article/10.3390/jcm15207898/s1. Figure S1: Participant selection flowchart; Figure S2: Longitudinal gait-cycle waveforms; Table S1: Longitudinal changes in the secondary gait outcomes estimated with linear mixed-effects models; Table S2: Additional repeated-measures ANOVA results for comparison with the linear mixed-effects models.
