# Supplementary material for: Longitudinal Course of Gait Kinematics and Kinetics After Guided Growth for Idiopathic Genu Valgum: Three-Dimensional Gait Analysis to Skeletal Maturity
Source: J Clin Med. 2026 Oct 13;15(20):7898. doi: 10.3390/jcm15207898 (PMC13706546; doi:10.3390/jcm15207898)
Supplement: Supplementary file 1 [file jcm-15-07898-s001.zip › jcm-4498337-supplementary.pdf]

Supplementary Material

Supplementary Table S1. Longitudinal changes in the secondary gait outcomes estimated with linear mixed-effects models.

Outcome	Contrast	Δ	Nominal 95% CI	Adjusted *p*
Walking speed, m/s	t1 − t0	+0.020	−0.045 to 0.085	1.000
	t2 − t1	+0.010	−0.055 to 0.075	1.000
	t2 − t0	+0.030	−0.035 to 0.095	1.000
Normalized walking speed	t1 − t0	0.000	−0.022 to 0.022	1.000
	t2 − t1	−0.010	−0.032 to 0.012	1.000
	t2 − t0	−0.010	−0.032 to 0.012	1.000
Cadence, steps/min	t1 − t0	−3.10	−6.19 to −0.01	0.149
	t2 − t1	−2.02	−5.11 to 1.07	0.556
	t2 − t0	−5.12	−8.21 to −2.03	**0.009**
Normalized cadence	t1 − t0	−0.010	−0.025 to 0.005	0.510
	t2 − t1	0.000	−0.015 to 0.015	1.000
	t2 − t0	−0.010	−0.025 to 0.005	0.510
Step width, m	t1 − t0	−0.0200	−0.0304 to −0.0096	**0.003**
	t2 − t1	+0.0100	−0.0004 to 0.0204	0.177
	t2 − t0	−0.0100	−0.0204 to 0.0004	0.177
Foot progression, °	t1 − t0	+0.33	−1.22 to 1.88	1.000
	t2 − t1	−1.01	−2.56 to 0.54	0.559
	t2 − t0	−0.68	−2.23 to 0.87	1.000
Hip rotation, °	t1 − t0	+1.85	−0.19 to 3.89	0.218
	t2 − t1	−0.57	−2.61 to 1.47	1.000
	t2 − t0	+1.28	−0.76 to 3.32	0.606
Knee rotation, °	t1 − t0	−1.43	−4.09 to 1.23	0.812
	t2 − t1	+2.63	−0.03 to 5.29	0.156
	t2 − t0	+1.20	−1.46 to 3.86	1.000
Frontal hip angle, early maximum, °	t1 − t0	−1.57	−2.64 to −0.50	**0.021**
	t2 − t1	+1.21	0.14 to 2.28	0.089
	t2 − t0	−0.36	−1.43 to 0.71	1.000
Frontal hip angle, late maximum, °	t1 − t0	−2.40	−3.57 to −1.23	**0.002**
	t2 − t1	+2.28	1.11 to 3.45	**0.002**
	t2 − t0	−0.12	−1.29 to 1.05	1.000
Internal frontal hip moment, early maximum, Nm/kg	t1 − t0	+0.060	0.018 to 0.102	**0.025**
	t2 − t1	+0.040	−0.002 to 0.082	0.186
	t2 − t0	+0.100	0.058 to 0.142	**<0.001**
Internal frontal hip moment, late maximum, Nm/kg	t1 − t0	+0.040	−0.008 to 0.088	0.281
	t2 − t1	+0.110	0.062 to 0.158	**<0.001**
	t2 − t0	+0.150	0.102 to 0.198	**<0.001**
Frontal knee range of motion, °	t1 − t0	+0.74	−0.62 to 2.10	0.794
	t2 − t1	−1.27	−2.63 to 0.09	0.194
	t2 − t0	−0.53	−1.89 to 0.83	1.000
Trunk lean, stance minimum, °	t1 − t0	−0.22	−0.88 to 0.44	1.000
	t2 − t1	+0.34	−0.32 to 1.00	0.881
	t2 − t0	+0.12	−0.54 to 0.78	1.000
Trunk lean, cycle mean, °	t1 − t0	+0.05	−0.55 to 0.65	1.000
	t2 − t1	+0.09	−0.51 to 0.69	1.000
	t2 − t0	+0.14	−0.46 to 0.74	1.000

Contrasts represent the later assessment minus the earlier assessment. Values are model estimates with nominal 95% CIs. The p values are Bonferroni-adjusted across the three time contrasts within each outcome. Bold p values indicate $p \leq 0.05$. Values were analyzed at the participant level for walking speed, cadence, their normalized measures, and step width (17 participants), and at the limb level for kinematics and kinetics (31 limbs from 17 participants). Limb-level models included participant and limb-within-participant random effects. All time contrasts used 16 degrees of freedom. t0, preoperative assessment; t1, shortly before plate removal; t2, final follow-up after skeletal maturity. Δ, estimated change; CI, confidence interval. Negative transverse-plane values indicate relative external rotation.

Supplementary Table S2. Additional repeated-measures ANOVA results for comparison with the linear mixed-effects models.

Outcome	Cohen's f	*p* t0–t1	*p* t1–t2	*p* t0–t2
Frontal knee angle, °	1.61	**<0.001**	0.540	**<0.001**
Knee moment, early maximum, Nm/kg	1.50	**<0.001**	1.000	**<0.001**
Knee moment, late maximum, Nm/kg	1.82	**<0.001**	0.437	**<0.001**
Knee moment, stance mean, Nm/kg	1.85	**<0.001**	1.000	**<0.001**
Walking speed, m/s	—	1.000	1.000	0.843
Normalized walking speed	—	1.000	1.000	1.000
Cadence, steps/min	—	0.076	0.385	**0.028**
Normalized cadence	—	0.727	1.000	1.000
Step width, m	—	**0.001**	0.196	0.510
Foot progression, °	—	1.000	0.392	1.000
Hip rotation, °	—	0.516	1.000	1.000
Knee rotation, °	—	1.000	0.092	1.000
Hip angle, early maximum, °	—	**0.004**	0.109	1.000
Hip angle, late maximum, °	—	**<0.001**	**<0.001**	1.000
Hip moment, early maximum, Nm/kg	—	**0.010**	0.090	**<0.001**
Hip moment, late maximum, Nm/kg	—	0.143	**<0.001**	**<0.001**
Frontal knee range of motion, °	—	1.000	0.138	1.000
Trunk lean, stance minimum, °	—	1.000	0.712	1.000
Trunk lean, cycle mean, °	—	1.000	1.000	1.000

Pairwise *p* values are Bonferroni-adjusted within each outcome. Cohen's f quantifies the overall time effect and was calculated for the four primary outcomes. Bold *p* values indicate $p \leq 0.05$.

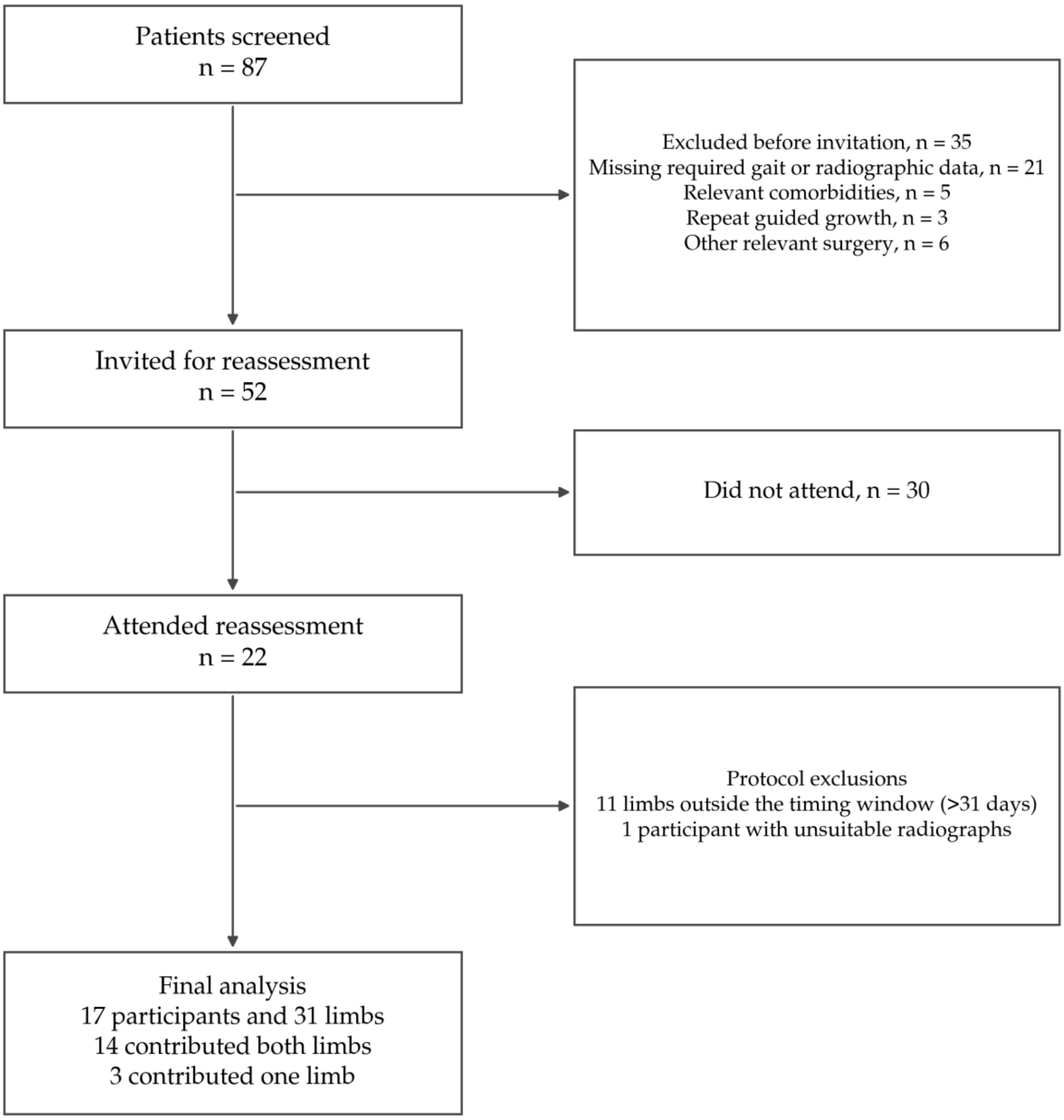

Supplementary Figure S1. Participant selection flowchart.

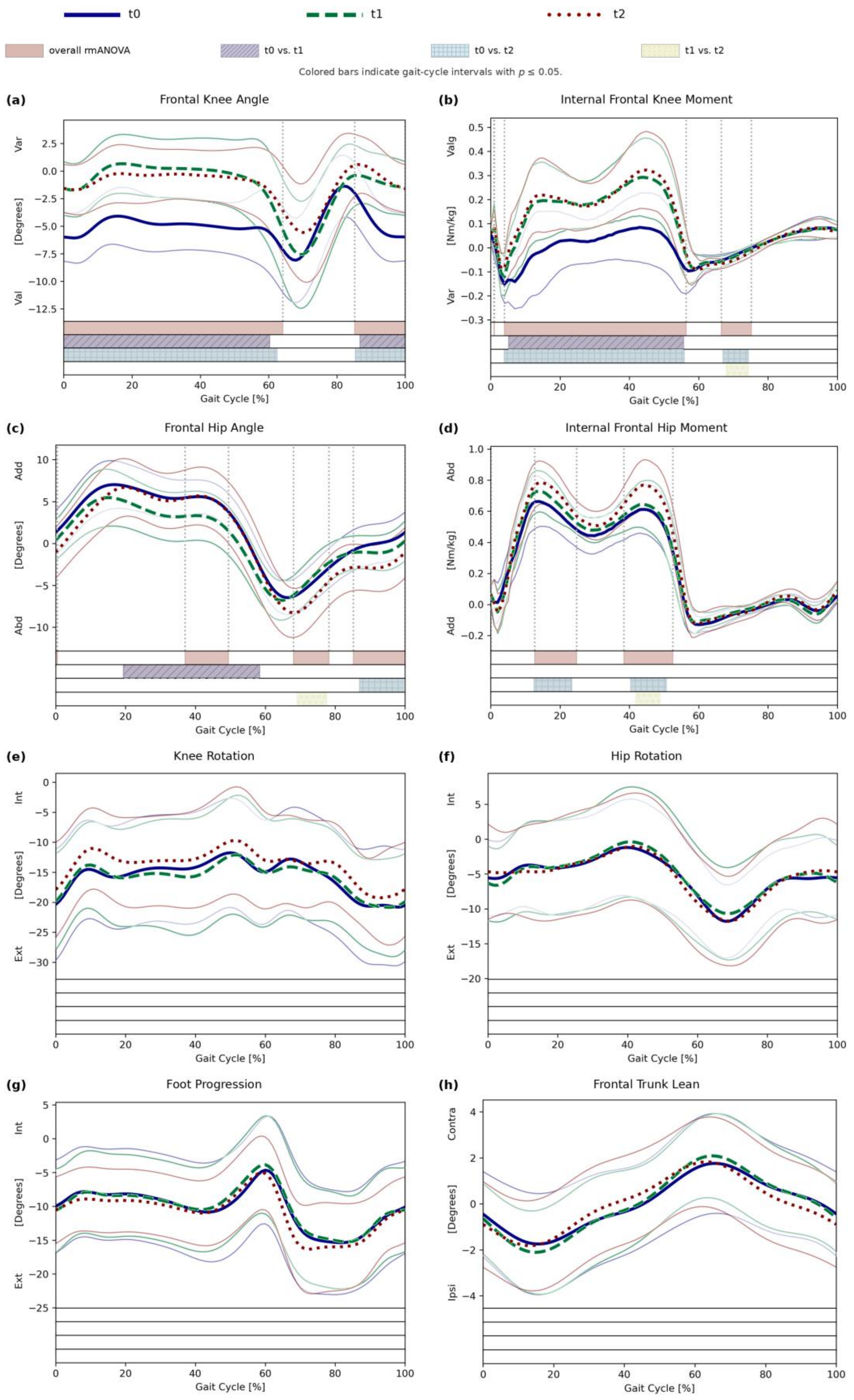

Supplementary Figure S2. Longitudinal gait-cycle waveforms: (a) frontal knee angle; (b) internal frontal knee moment; (c) frontal hip angle; (d) internal frontal hip moment; (e) knee rotation; (f) hip rotation; (g) foot progression; and (h) frontal trunk lean. Dark-blue solid, dark-green dashed, and dark-red dotted lines represent t0, t1, and t2; thick lines show means and thin lines ±1 SD. Colored bars identify significant SPM gait-cycle regions ($p \leq 0.05$): pink, overall time effect across t0, t1, and t2 within the study cohort; purple hatched, t0–t1; blue crosshatched, t0–t2; yellow patterned, t1–t2.
